# Identifying excitatory and inhibitory synapses in neuronal networks from dynamics using Transfer Entropy

**DOI:** 10.1186/1471-2202-16-S1-P30

**Published:** 2015-12-18

**Authors:** Felix Goetze, Pik-Yin Lai, CK Chan

**Affiliations:** 1 Department of Physics, National Central University, Chung-Li, Taiwan, Republic of China; 2 Taiwan International Graduate Program for Molecular Science and Technology, Institute for Atomic and Molecular Sciences, Academia Sinica, Taipei, Taiwan, Republic of China; 3 Institute of Physics, Academia Sinica, Taipei, Taiwan, Republic of China

## 

Measuring the effective connectivity between the elements of a neuronal system promises to give conclusions about brain function and the principles of information processing in the brain. Transfer Entropy [1] (TE) and its extensions have recently become more and more popular for this task and have been applied to various types of neuronal data, such as from EEG, Calcium Imaging and Multi-electrode Array measurements [2]. Being based on information theory, TE can be interpreted as the predictive information transfer between two time series. TE is a model free measurement and quantifies even non-linear interactions and very importantly the directionality.

However, due to its information-theoretic nature, TE doesn't distinguish between different types of interactions, for example whether a pre-synaptic neuron drives a post-synaptic neuron via an excitatory or an inhibitory synapse [3]. This distinction is crucial for the understanding of the network dynamics and the exact interplay of excitation and inhibition in neuronal networks plays an important role for network bursts and synchronization. The balance of excitation and inhibition is believed to play a role for the occurrence of epileptic seizures [4].

We propose a method complementary to the TE measurement, to not only measure the effective connectivity from neuronal network dynamics, but also to classify, whether the interactions are excitatory or inhibitory. We achieve this by introducing a new quantity, which is a linear combination of the individual terms that sum up to the Transfer Entropy. This quantity can be computed by applying a Principal Component Analysis (PCA) to the Transfer Entropy terms across different measurements. It assumes positive values for excitatory connections and negative values for inhibitory connections.

To verify this method we apply it to the spike trains of noise-driven recurrent neuronal network motifs, simulated with an extended Fitzhugh-Nagumo model. We analyze all possible combinations of three neurons being coupled with either excitatory or inhibitory synapses, which add up to a total of 132 network motifs. The best performance of our reconstruction of these motifs, being measured by the area under the Receiver Operating Characteristic curve, exceeds values of 0.99. Together with the PCA analysis, we are not only able to reconstruct the motif topologies from the neuronal dynamics, but we are also able to distinguish between excitatory and inhibitory synapses.
